# Fabrication of a paper-based colorimetric molecular test for SARS-CoV-2

**DOI:** 10.1016/j.mex.2021.101586

**Published:** 2021-11-20

**Authors:** Jiangshan Wang, Andres Dextre, Ana Pascual-Garrigos, Josiah Levi Davidson, Murali K. Maruthamuthu, Darby McChesney, Jordan Seville, Mohit S. Verma

**Affiliations:** a Department of Agricultural and Biological Engineering, Purdue University, West Lafayette, IN 47907, USA; b Birck Nanotechnology Center, Purdue University, West Lafayette, IN 47907, USA; c PortaScience, Inc., Moorestown, NJ 08057, USA; d Weldon School of Biomedical Engineering, Purdue University, West Lafayette, IN 47907, USA

**Keywords:** Paper-based diagnostics, SARS-CoV-2, Colorimetric LAMP, Microfluidic paper-based analytical devices

## Abstract

The ongoing pandemic caused by severe acute respiratory syndrome coronavirus 2 (SARS-CoV-2) has caused unprecedented damage to the global economy. Diagnostic testing is a key factor in limiting virus transmission and safeguarding public health. We present the fabrication process of a paper-based device that uses reverse-transcription loop-mediated isothermal amplification (RT-LAMP) to detect SARS-CoV-2 in complex matrices by providing a colorimetric response apparent to the naked eye. Because of LAMP's functionality, this device just requires a simple heat source (e.g., water bath, incubator), it can be deployed in resource-constrained areas and can be used as a supplement to current point-of-care (POC) and community testing procedures. Since the test is based on nucleic acids, the testing platform itself lends to further applications including food safety monitoring, animal diagnostics, etc. simply by changing the specific primers.

• We developed a platform capable of on-paper detection of SARS-CoV-2 using colorimetric reporters that produce responses visible to the naked eye.
• The platform is easily reconfigurable to target different pathogens by changing the primer sets, and multiplexing is possible by adding additional reaction sites to the device.

## Graphical abstract

**Figure:**
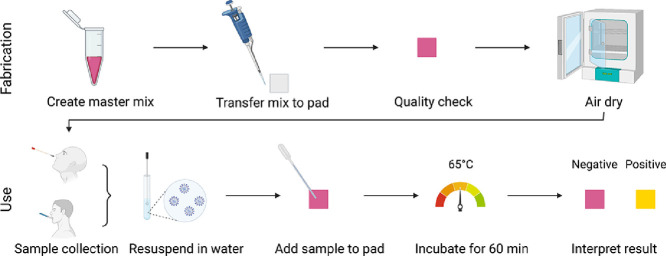

Specifications table

**Table:** 

Subject Area:	Engineering
More specific subject area:	Biosensors, Microfluidic paper-based analytical devices
Method name:	Paper-based colorimetric LAMP
Name and reference of original method:	N/A
Resource availability:	N/A

## Method details

Herein, we present the fabrication of a paper-based device that uses loop-mediated isothermal amplification (LAMP) to detect nucleic acids of pathogens of interest in complex samples by providing a colorimetric response observable to the naked eye. We have previously demonstrated that this device can be used to detect SARS-CoV-2 in human saliva with a limit of detection of 200 copies/µL saliva [1]. Unlike most nucleic-acid-based biosensors for SARS-CoV-2 detection [2,3,4], this device does not require prior RNA extraction or concentration. The paper-based colorimetric LAMP assay builds upon previous work of liquid-based colorimetric LAMP assay [5]. Although many of the components are available as a part of a LAMP Master Mix (M1804 from New England Biolabs), the concentrations of the reagents in the LAMP Master Mix (M1804) are not optimized for paper-based reactions and result in low-contrast results [1]. The paper-based approach was inspired by a previous demonstration where hydroxynaphtol blue was used as a fluorescent reporter in a paper-based device [6]. The technique of long-term dry storage of LAMP reagents was adapted from a previous study that highlighted the role of excipients in storage of enzymes [7]. Due to the simplicity and scalability of this device, it can be used in a variety of applications, enabling the detection of nucleic acids from pathogens in a portable field-deployable manner.

Required reagents:

Potassium chloride (P9541, Sigma-Aldrich, USA), Magnesium sulfate (M2773, Sigma-Aldrich, USA), Deoxynucleotide triphosphate (dNTP) (N0447, New England Biolabs, USA), *Bst* 2.0 DNA Polymerase (M0537M, New England Biolabs, USA), WarmStart® RTx Reverse Transcriptase (M0380S, New England Biolabs, USA), Phenol red (P3532, Sigma-Aldrich, USA), Deoxyuridine Triphosphate (dUTP) (FERR0133, Thermo Scientific, USA), Antarctic Thermolabile UDG (M0372S, New England Biolabs, USA), Polysorbate 20 (278632500, Thermo Scientific Chemicals, USA), Betaine (B0300-5VL, Sigma-Aldrich, USA), Bovine serum albumin (optional) (A2153, Sigma-Aldrich, USA), Trehalose (182550250, Thermo Scientific Chemicals, USA), Nuclease-Free Water (4387936, Thermo Scientific, USA), RNase AWAY (optional) (147002, Research Products International Corp, USA).

The catalog numbers listed are the reagents we used in our study. The same reagents from alternative sources are likely to function well too.

Required equipment:

Forceps, 0.5-10 µL Pipette, 2-20 µL Pipette, 20-200 µL Pipette, 100-1000 µL Pipette, Ahlstrom-Munksjö Grade 222 (22282020, Ahlstrom-Munksjo, Finland), pH probe, a heat source that could reach 65°C (e.g., incubator, water bath), PCR hood (optional)

Before you start:

• Spray pipettes and all workbench (PCR hood) surfaces with RNase AWAY. Wipe thoroughly after applying RNase AWAY. It will interfere with the reaction if there is any remaining.
• It is preferable to have separate rooms for fabricating the paper-based device and loading the samples. This will aid in the prevention of cross-contamination.
• Pre-cut 5 mm x 6 mm chromatography paper (Ahlstrom-Munksjö Grade 222).

LAMP preparation:

1. Prepare 2X LAMP mix as indicated in the chart below inside a PCR hood.

**Table:** 

2X LAMP Mix						

Components	Volume	Unit	Stock concentration	Unit	Final concentration	Unit

KCL	100	µL	1000	mM	100	mM
MgSO_4_	160	µL	100	mM	16	mM
dNTPs	280	µL	10	mM	2.8	mM
*Bst* 2.0 DNA Polymerase	10.8	µL	120	U/µL	1.296	U/µL
RTx Reverse Transcriptase	40	µL	15	U/µL	0.6	U/µL
Phenol red	20	µL	25	mM	0.2	mM
dUTPs	2.8	µL	100	mM	0.28	mM
Antarctic Thermolabile UDG	0.4	µL	1	U/µL	0.0004	U/µL
Polysorbate 20	100	µL	20	%	2	%
Nuclease-Free Water	286	µL	-	-	-	-
Total					1000	µL

2. Adjust pH with 1M KOH (approximately 1-2 µL) to pH ∼7.5–8.0 (a red but not pink color). Does not need to be precise. After adjusting pH, 2X LAMP Mix could be stored at -20°C.

**Table:** 

Complete mix for paper LAMP						

Components	Volume	Unit	Stock concentration	Unit	Final concentration	Unit

2X LAMP Mix	125	µL	-	-	-	-
10X Primer Mix^a^	25	µL	-	-	-	-
Betaine	1	µL	5	M	20	mM
BSA (optional)	3.13	µL	40	mg/mL	0.626	mg/mL
Trehalose	36	µL	689	mg/mL	-	-
Water	9.2	µL				
Total					200	µL

a We recommend making stocks of the LAMP primers at a workable concentration in water for ease of setup. We suggest making a 10X Primer Mix containing all 6 LAMP primers.10X Primer mix: 16 µM FIP/BIP, 2 µM F3/B3, 4 µM Loop F/B.

3. Prepare master mix according to the chart below.

1. Adjust pH to 8.0 with 0.1M KOH. We recommend using a micro-pH electrode (e.g., 11-747-328, Fisher Scientific, USA)
2. Mix thoroughly. Lay paper pads out on a clean surface inside the PCR hood. Add 30 µL of complete mix on pre-cut grade 222 paper pads.
3. Dry under PCR hood at room temperature for 60 min.
4. After drying, collect paper pads into a clean centrifuge tube (e.g., 05-408-129, Fisher Scientific, USA) or a clean re-sealable plastic bag (e.g., S-12250, Uline, USA).

Sample loading:

1. Spray down the working bench with RNase AWAY and clean it with wipes.
2. Take out templates (DNA, RNA, heat-inactivated virus) from the freezer.
3. Lay out the reaction pads on a clean surface. You can lay your pads on a new transparency film (617993, Office Depot, USA) (optional) and discard it after use.
4. Prepare negative control pads first. Reconstitute pads with 25 µL non-template solvent (water, saliva). The reconstitution process should be gentle, avoid washing out the regents from the pad.
5. Place the negative control pads inside a clean container (e.g., 1” x 1” resealable plastic bag, centrifuge tube) using forceps.
6. Dilute template (if necessary) with solvent into desired concentration.
7. Lay out more reaction pads and reconstitute pads with 25 µL diluted template.
8. Place the positive pads inside a clean container (e.g., 1” x 1” resealable plastic bag, centrifuge tube) using forceps.
9. Clean up the workspace and bring the pads for imaging and incubating.

Imaging and incubation:

Note: There are multiple imaging methods (e.g., time-lapse video, scanning) and heat sources (e.g., incubator, water bath). In this protocol, we will be using a tabletop scanner and a microbiological incubator.

1. Arrange the pads on top of the scanner. Scan the pads before the reaction (0 min).
2. Preheat the incubator to 65°C.
3. Place the pads into the incubator. Make sure they are well separated. The heating uniformity would affect the result consistency.
4. Take out pads and repeat scanning at different time points (usually every 30 min).
5. After final scanning, discard the reaction pads inside biohazard trash.

## Method validation

**Fig. 1:**
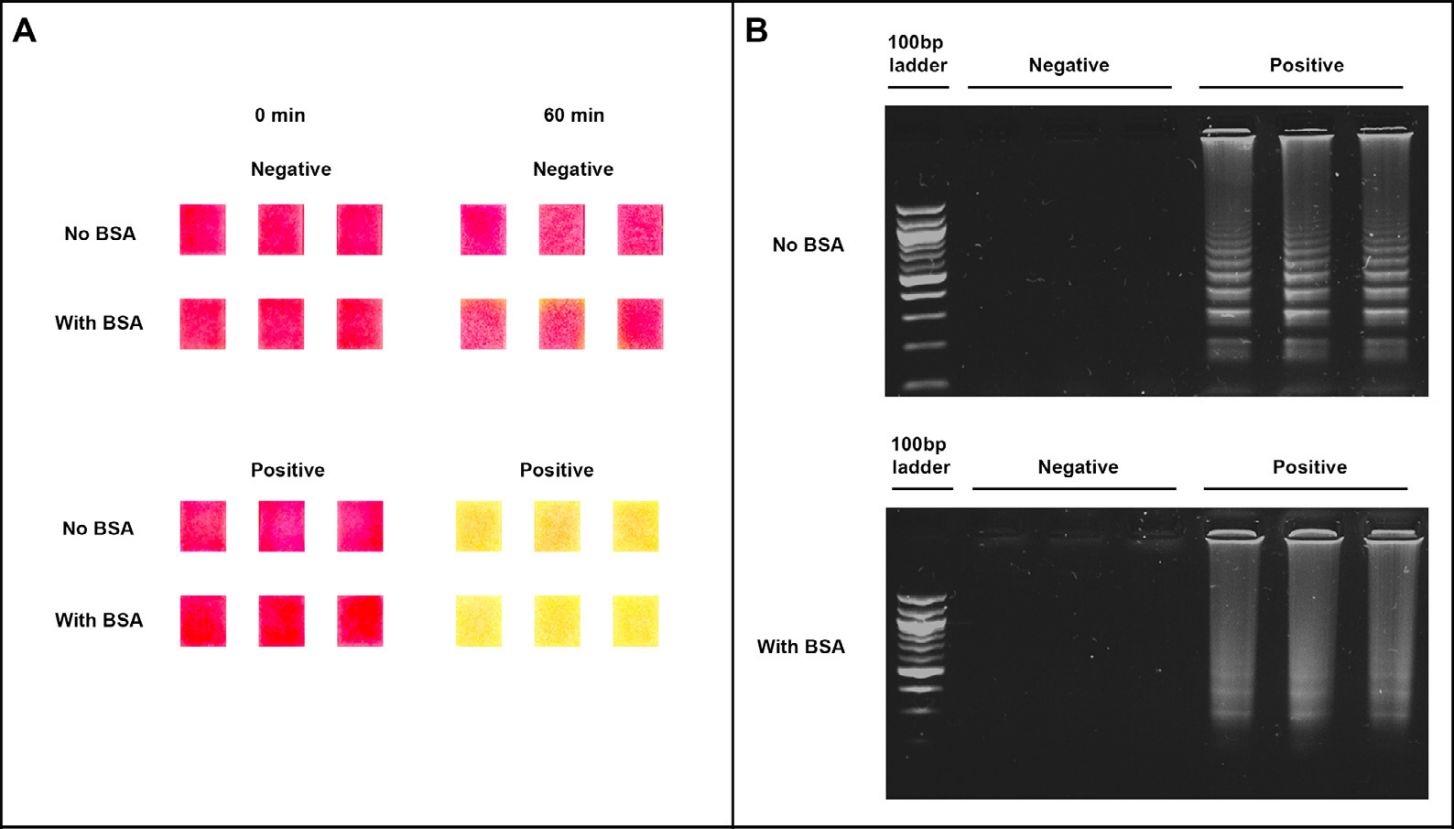
Paper LAMP validation. (A) LAMP on paper with two conditions (with and without BSA in the reaction mix). (B) associated results from gel electrophoresis (2% agarose). The orf7ab.1 primer set targeting SARS-CoV-2 was used. Negative reaction pads were reconstituted with 25 µL nuclease free water. Positive reaction pads were reconstituted with 25 µL 400 copies/µL heat-inactivated SARS-CoV-2 virus (VR-1986HK, ATCC, USA). Heating was carried out in an incubator set at 65°C and scanned in a flatbed scanner.

To verify the occurrence of LAMP amplification, each reaction pad was transferred to a clean 1.5 mL microcentrifuge tube. 100 µL Buffer EB (19086, Qiagen, Germany) was added to each tube. Reaction pads were submerged in Buffer EB overnight for eluting nucleic acids. Gel electrophoresis (2% agarose gel) was performed with the eluent to verify the occurrence of LAMP amplification. A ladder-like pattern (typical LAMP product pattern) was shown in each positive pad lane while there was no obvious band in each negative lane (Fig. 1).

**Fig. 2:**
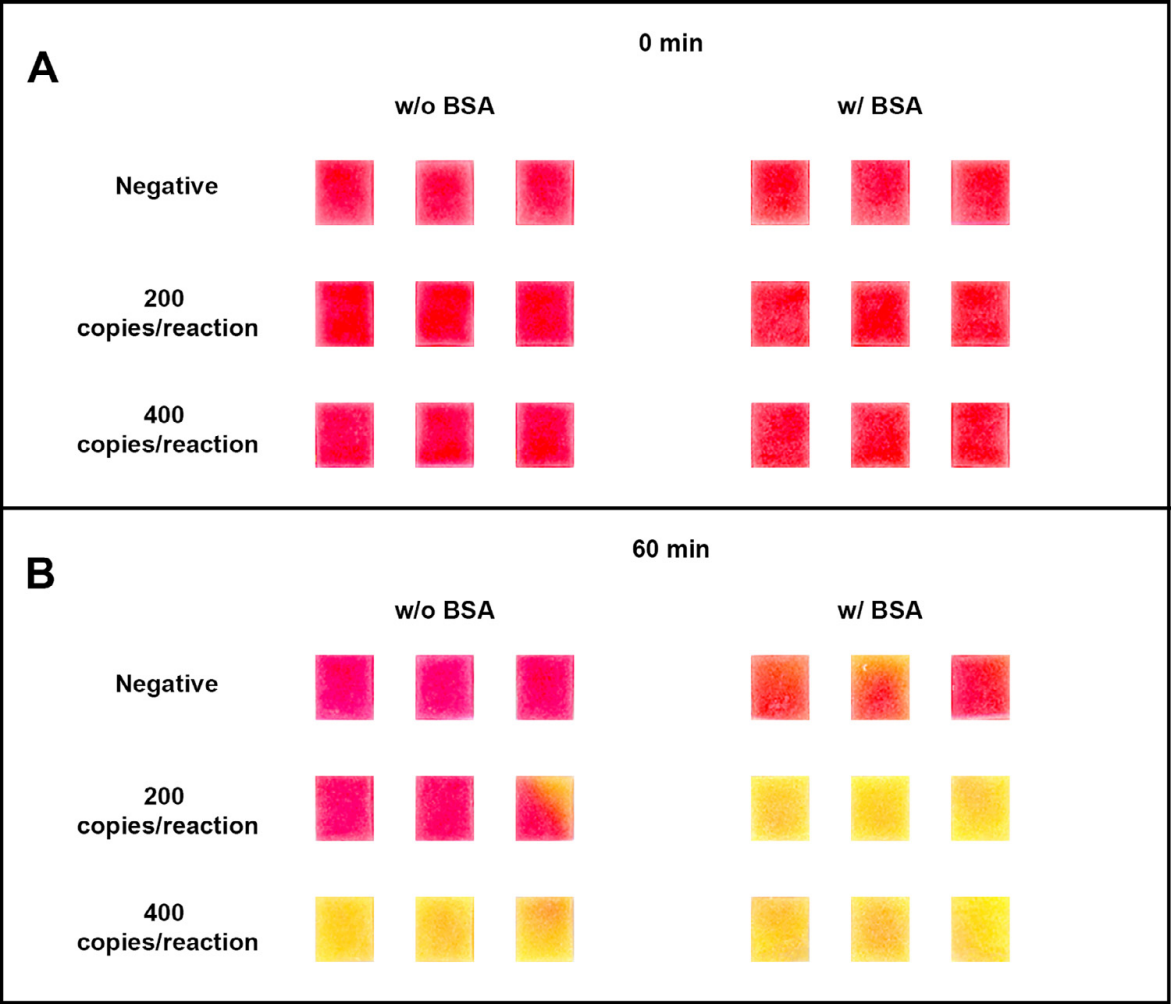
Low template concentration LAMP on paper with two conditions (with and without BSA in the reaction mix). (A) 0 min time point. (B) 60 min time point. The orf7ab.1 primer was used in this experiment. Negative reaction pads were reconstituted with 25 µL nuclease free water. Positive reaction pads were reconstituted with 25 µL 8 copies/µL and 16 copies/µL heat-inactivated SARS-CoV-2 virus (VR-1986HK, ATCC, USA) (final concentration of 200 copies/reaction and 400 copies/reaction) respectively.

BSA is an optional reagent in the LAMP mix. Adding BSA will speed up reactions and improve sensitivity (Fig. 2), however, it will also introduce pH variations to the device. Figs. 1A and 2B show that after incubation (60 min) negative paper pads containing BSA will have a yellowish edge. After elution and running the eluent with gel electrophoresis, there was no DNA product visible on the gel (Fig. 1B). This indicates that the yellow color at the edge is not caused by off-target amplification or contamination. We speculate that a heterogeneous distribution of BSA leads to the yellow color at the edges upon application of heat.

## Troubleshooting

### Unusual pink color on paper pads

During the process of preparing LAMP paper pads, you might see unusual pink spots obviously different from the surrounding color. This might be because of residual RNase AWAY contacted either directly sprayed onto the pads and/or transferred via the forceps. RNase AWAY will degrade any RNA/DNA template added. If this problem was encountered, we recommend that you thoroughly dry all equipment and surfaces, cut new 5 × 6 mm paper pads, and restart the ‘LAMP preparation’ section from Step 5.

### Overflowing of reagents after pad reconstitution

During the sample loading step, you might see the pad is unable to absorb the entire sample volume added to it for reconstitution. The template concentration will not be accurately represented by pads that overflow. Overflowing can be caused by insufficient drying of the pads. If encountered with this problem, we recommend to: 1) dry for a longer time, 2) use an enhanced drying method such as heat drying (place on a clean microbiological incubator at 37°C; do NOT set the temperature higher than 45°C to prevent activation of the *Bst* 2.0 WarmStart® polymerase) or convective drying (use small fans to enhance airflow during drying), or 3) reduce reconstitution volume to 20 µL.

### Negative controls exhibit color change

During the imaging and incubation step, you might encounter the negative pads changing at the same time or shortly after the sample-containing pads. This issue can be caused by either primer dimerization/non-specific amplification or carryover contaminants of previous LAMP reactions. We recommend validating the primer set in liquid-based LAMP prior to using them on paper. To control carryover contamination, we recommend the following: (1) implement dUTPs and UDG in all LAMP reactions, (2) maintain separate working stations for LAMP mixture preparation and sample addition, and (3) aliquot reagent stocks and use new aliquots if contamination is suspected to have occurred. Over-incubating the reaction would also induce non-specific amplification. We recommend not to exceed an incubation time of 75 min.

### Sample pH and buffering capacity will influence colorimetric readouts

Since phenol red is a pH indicator, the pH of the sample and its buffering capacity will have a significant impact on the assay. We have confirmed that saliva concentrations of 5-10% v/v (diluted with water) work with the reagent composition presented here [1]. We chose 5% saliva because it has a faster response time and produces more consistent results. Human saliva has a sophisticated buffering system that includes bicarbonate salts, phosphate salts, and proteins, which prevents pH change (and thus color change) at high saliva concentrations. We have also tested the paper LAMP device with nasal swab resuspended in water and we did not observe any inhibition from the sample matrix. Colorimetric readouts are also likely to be hampered by buffered salt solution (e.g., transport media).

## Direct submission or co-submission

*Co-Submission* 10.1016/j.biosx.2021.100076

## References

[1] Davidson J.L., Wang J., Maruthamuthu M.K., Dextre A., Pascual-Garrigos A., Mohan S., Putikam S.V.S., Osman F.O.I., McChesney D., Seville J., Verma M.S. (2021). A paper-based colorimetric molecular test for SARS-CoV-2 in saliva. Biosens. Bioelectron. X.

[2] Broughton J.P., Deng X., Yu G., Fasching C.L., Servellita V., Singh J., Miao X., Streithorst J.A., Granados A., Sotomayor-Gonzalez A., Zorn K., Gopez A., Hsu E., Gu W., Miller S., Pan C.-Y., Guevara H., Wadford D.A., Chen J.S., Chiu C.Y. (2020). CRISPR–Cas12-based detection of SARS-CoV-2. Nat. Biotechnol..

[3] Joung J., Ladha A., Saito M., Kim N.-G., Woolley A.E., Segel M., Barretto R.P.J., Ranu A., Macrae R.K., Faure G., Ioannidi E.I., Krajeski R.N., Bruneau R., Huang M.-L.W., Yu X.G., Li J.Z., Walker B.D., Hung D.T., Greninger A.L., Zhang F. (2020). Detection of SARS-CoV-2 with SHERLOCK One-Pot Testing. N. Engl. J. Med..

[4] Yamazaki W., Matsumura Y., Thongchankaew-Seo U., Yamazaki Y., Nagao M. (2021). Development of a point-of-care test to detect SARS-CoV-2 from saliva which combines a simple RNA extraction method with colorimetric reverse transcription loop-mediated isothermal amplification detection. J. Clin. Virol..

[5] Tanner N.A., Zhang Y., Evans T.C. (2015). Visual detection of isothermal nucleic acid amplification using pH-sensitive dyes. BioTechniques.

[6] Seok Y., Joung H.A., Byun J.Y., Jeon H.S., Shin S.J., Kim S., Shin Y.B., Han H.S., Kim M.G. (2017). A paper-based device for performing loop-mediated isothermal amplification with real-time simultaneous detection of multiple DNA targets. Theranostics.

[7] Kumar S., Gallagher R., Bishop J., Kline E., Buser J., Lafleur L., Shah K., Lutz B., Yager P. (2020). Long-term dry storage of enzyme-based reagents for isothermal nucleic acid amplification in a porous matrix for use in point-of-care diagnostic devices. Analyst.

